# Vestibular Paroxysmia: A Four-Case Report

**DOI:** 10.7759/cureus.71930

**Published:** 2024-10-20

**Authors:** Elizabeth G Saenz Zapata, Monica P Alcantara Thome, Blanca Isabel Perez Hernandez

**Affiliations:** 1 Audiology and Otoneurology, Hospital de Especialidades del Centro Médico Nacional Siglo XXI, Instituto Mexicano del Seguro Social (IMSS), Mexico City, MEX; 2 Audiology and Otoneurology, Instituto Mexicano del Seguro Social (IMSS), Mexico City, MEX

**Keywords:** neurovascular compression, peripheral vertigo, positional vertigo, recurrent dizziness, vestibular paroxysmia

## Abstract

Vestibular paroxysmia (VP) is a rare vestibular syndrome classified as a neurovascular compression disorder. It arises from chronic compression of the vestibulocochlear nerve caused by a vascular loop, leading to demyelination via ephaptic transmission. The diagnosis primarily relies on a thorough medical history that meets specific criteria. The differentiation between definite and probable VP is based on the response to medications such as carbamazepine or oxcarbazepine.

In this report, we present four patients over the age of 40 who experienced recurrent, brief episodes of vertigo, either spontaneously or triggered by head movements. These patients had undergone various treatments without success. Subsequent MRI scans revealed neurovascular compression of the VIII cranial nerve, prompting the prescription of oxcarbazepine, which resulted in a positive response in all cases. Although the differential diagnoses for these symptoms are limited, the rarity of VP can complicate diagnosis. Therefore, a trial of treatment should be considered in appropriate cases.

## Introduction

Vestibular paroxysmia (VP) is a rare condition; it had a prevalence of approximately 4% among patients in a study conducted at a specialized vertigo and dizziness care center [1]. The clinical manifestations are due to neurovascular compression of the eighth cranial nerve [2]. The primary symptoms include recurrent, brief episodes of spinning or non-spinning vertigo, typically lasting less than one minute [3]. While 60% of patients experience spontaneous attacks, they can also be triggered by head movements [4].

The diagnosis of VP or probable vestibular paroxysmia (PVP) primarily depends on the patient's medical history and specific diagnostic criteria. For VP, the criteria include at least 10 episodes of spontaneous spinning or non-spinning vertigo lasting less than one minute, a stereotyped phenomenology in a particular patient, response to treatment with carbamazepine or oxcarbazepine, and the absence of a better explanation from another diagnosis. In contrast, PVP is characterized by at least five episodes of spinning or non-spinning vertigo lasting less than five minutes, which may occur spontaneously or be triggered by certain head movements, with a stereotyped pattern in the patient and no better alternative diagnosis [5].

Clinical assessment remains crucial for diagnosing VP. However, MRI can be useful for ruling out central causes, as vascular loops are common anatomical variants that may or may not contribute to audiovestibular symptoms [6]. Vestibular paroxysmia is a treatable condition, and medical treatment with a low dose of oxcarbazepine is often effective [4]. This report presents four cases of challenging-to-diagnose, long-term vertigo attacks in adults that fulfilled the diagnostic criteria.

## Case presentation

Case one

**Figure 1:**
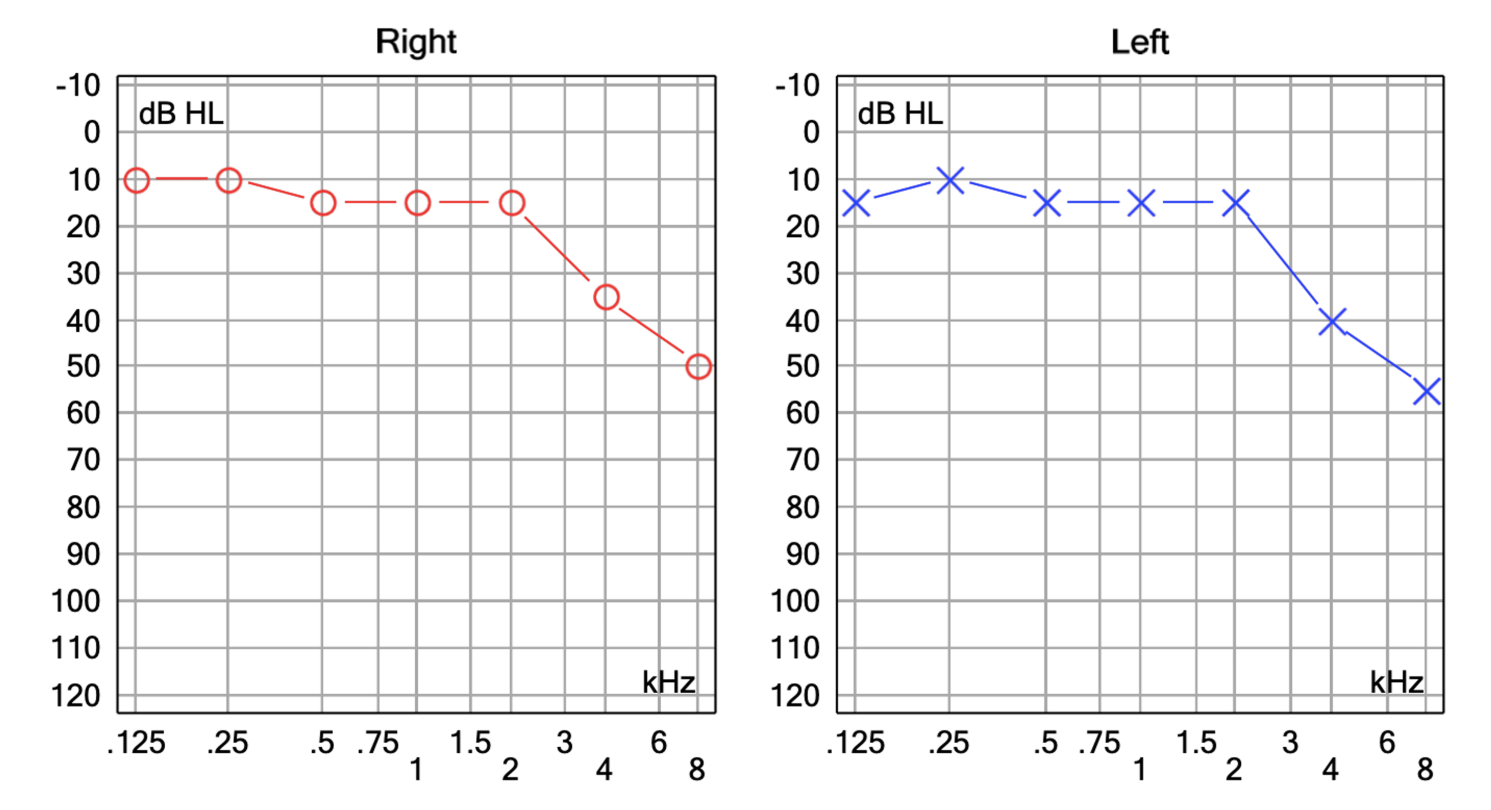
Pure tone audiometry of patient no.1

**Figure 2:**
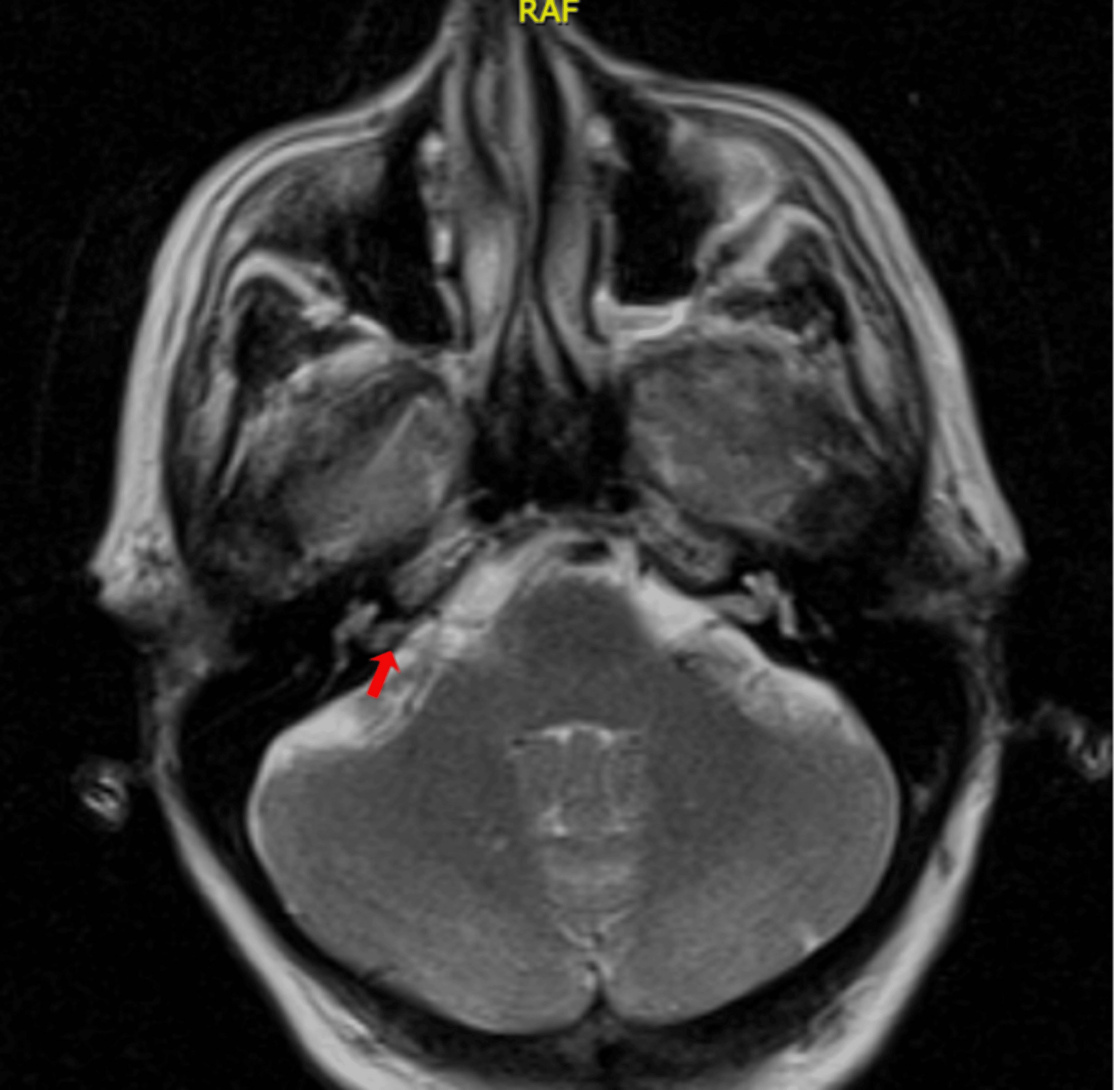
Axial T2-weighted brain MRI of patient no.1. Red arrow: Right-sided neurovascular compression (Chavda classification grade I)

A 62-year-old woman, with no personal medical history, attended the audiology and otoneurology department due to long-term episodic vertigo (38 years) of daily appearance, lasting seconds, related to head position, as well as nausea, vomiting, and unsteadiness. Previous treatment with diphenidol and cinnarizine was given with no response. Pure-tone audiometry showed high-frequency hearing loss (Figure 1). The vestibular examination showed a positional nystagmus mimicking anterior and/or posterior semicircular canal affection without a reversed direction after returning the patient to the upright position. Epley and Yacovino maneuvers were performed without resolving the nystagmus. Subsequent laboratory tests revealed a low vitamin D level of 16.27 mg/dL; however, supplementation was not initiated as an alternative etiology beyond benign paroxysmal positional vertigo (BPPV) was suspected. Imaging studies were conducted. A temporal bone CT scan showed normal findings. The brain MRI revealed a vascular loop compressing the right vestibulocochlear nerve located only at the cerebellopontine angle without entering the internal auditory canal (IAC); Chavda classification grade I (Figure 2). The patient was prescribed oxcarbazepine 300 mg/day, obtaining a great response as a follow-up examination one month later revealed a shorter duration of the positional nystagmus and less intensity and frequency of the vertigo attacks. The dizziness handicap inventory (DHI) was administered prior to starting oxcarbazepine and again one month later. The patient’s condition improved from 'severe disability' to 'mild disability.' The results are presented in Table 1.

**Table 1:** The DHI scores of patient no.1 DHI: Dizziness handicap inventory

Subscale	Pre-treatment score	Post-treatment score
Functional	26	10
Emotional	30	16
Physical	20	8
Total	76	34
Category	Severe	Mild

Case two

**Figure 3:**
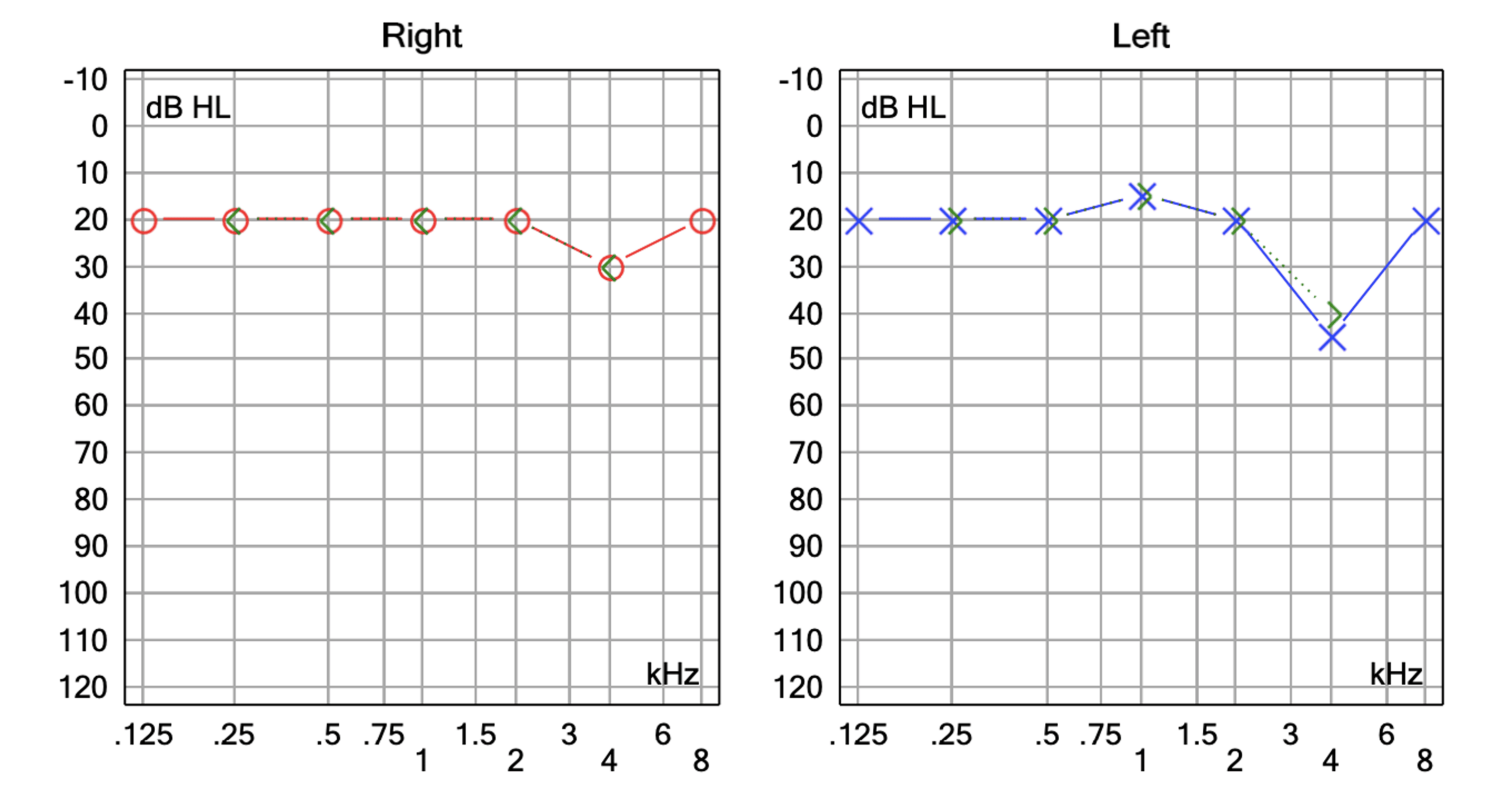
Pure tone audiometry of patient no.2

**Figure 4:**
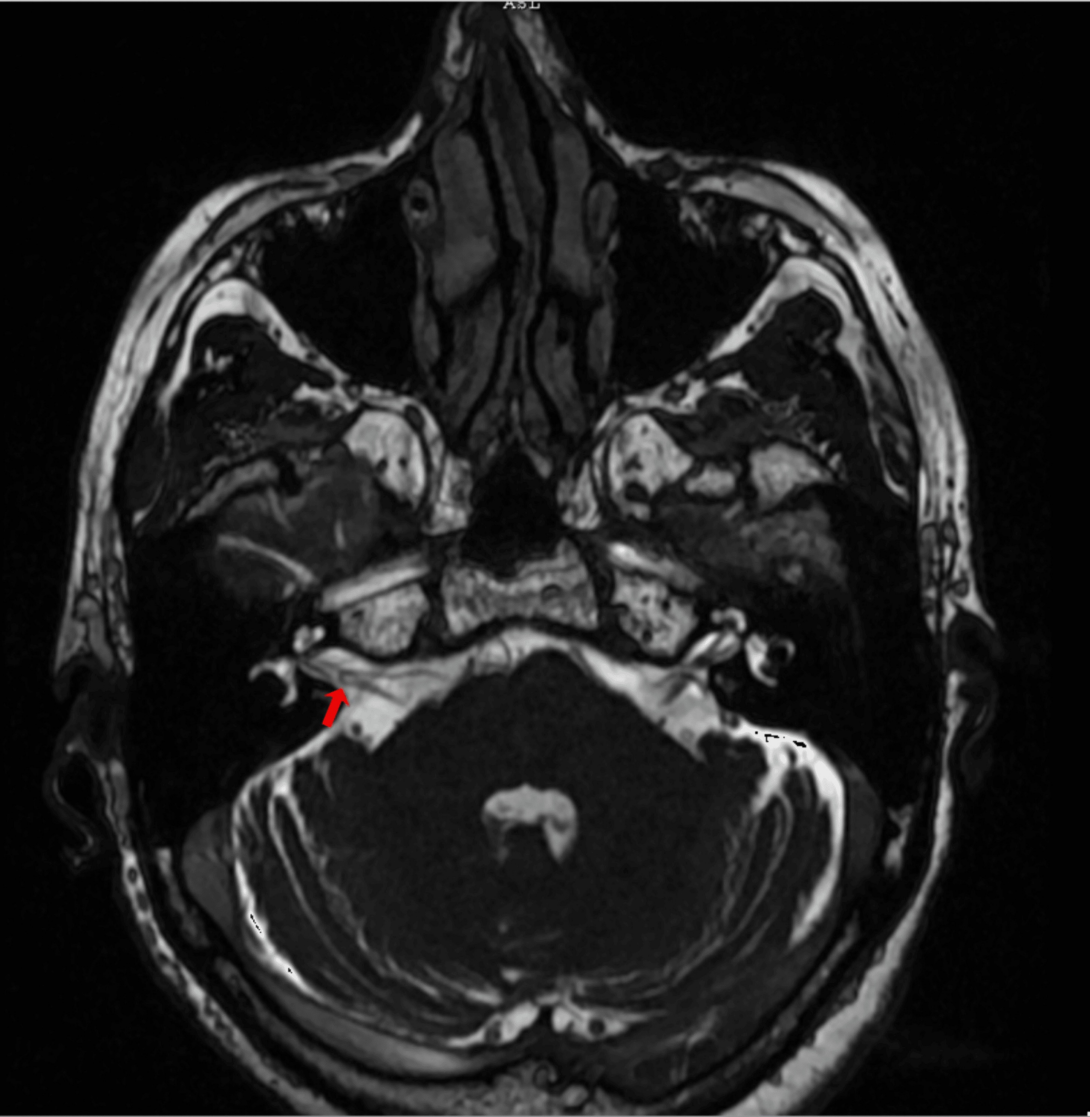
Axial FIESTA brain MRI of patient no.2 Red arrow: Right-sided neurovascular compression (Chavda classification grade II) FIESTA: Fast imaging employing steady-state acquisition

A 71-year-old woman with no personal medical history attended the audiology and otoneurology department due to 10-month unsteadiness and lateral displacement to the left, lasting seconds and sometimes triggered by head movements. Pure tone audiometry reported normal hearing with a bilateral typical noise-induced 4 kHz notch (Figure 3). Vestibular examination showed abnormal right clinical head impulse test and head shaking-induced horizontal nystagmus beating to the left. The patient underwent vestibular physical therapy over a month with gaze stabilization X1 and X2 paradigms without improvement. The fast imaging employing steady-state acquisition (FIESTA) sequence brain MRI revealed a right vascular loop contacting the cisternal portion of the acoustic-facial bundle and a slight increase in its diameter and was classified Chavda grade II as it enters, but does not extend more than 50% of the length of the IAC (Figure 4). Daily 300 mg oxcarbazepine was prescribed, with significant symptom improvement. The DHI was administered before initiating oxcarbazepine and again one month later. An improvement was observed, with the patient progressing from 'severe disability' to 'mild disability.' The results are displayed in Table 2.

**Table 2:** The DHI scores of patient no.2 DHI: Dizziness handicap inventory

Subscale	Pre-treatment score	Post-treatment score
Functional	34	8
Emotional	32	8
Physical	24	6
Total	90	22
Category	Severe	Mild

Case three

**Figure 5:**
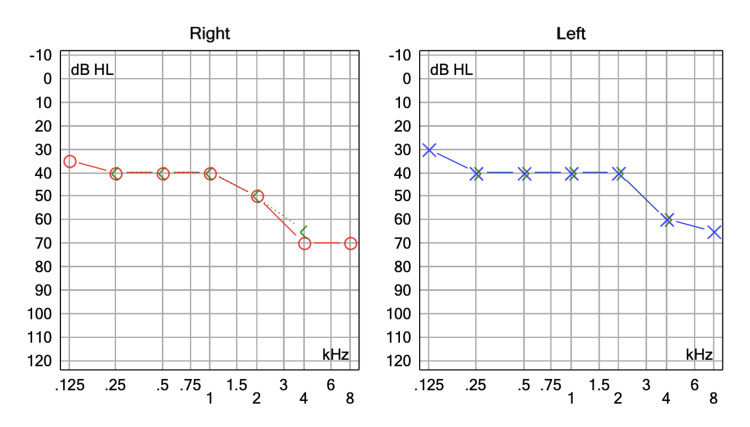
Pure tone audiometry of patient no.3

**Figure 6:**
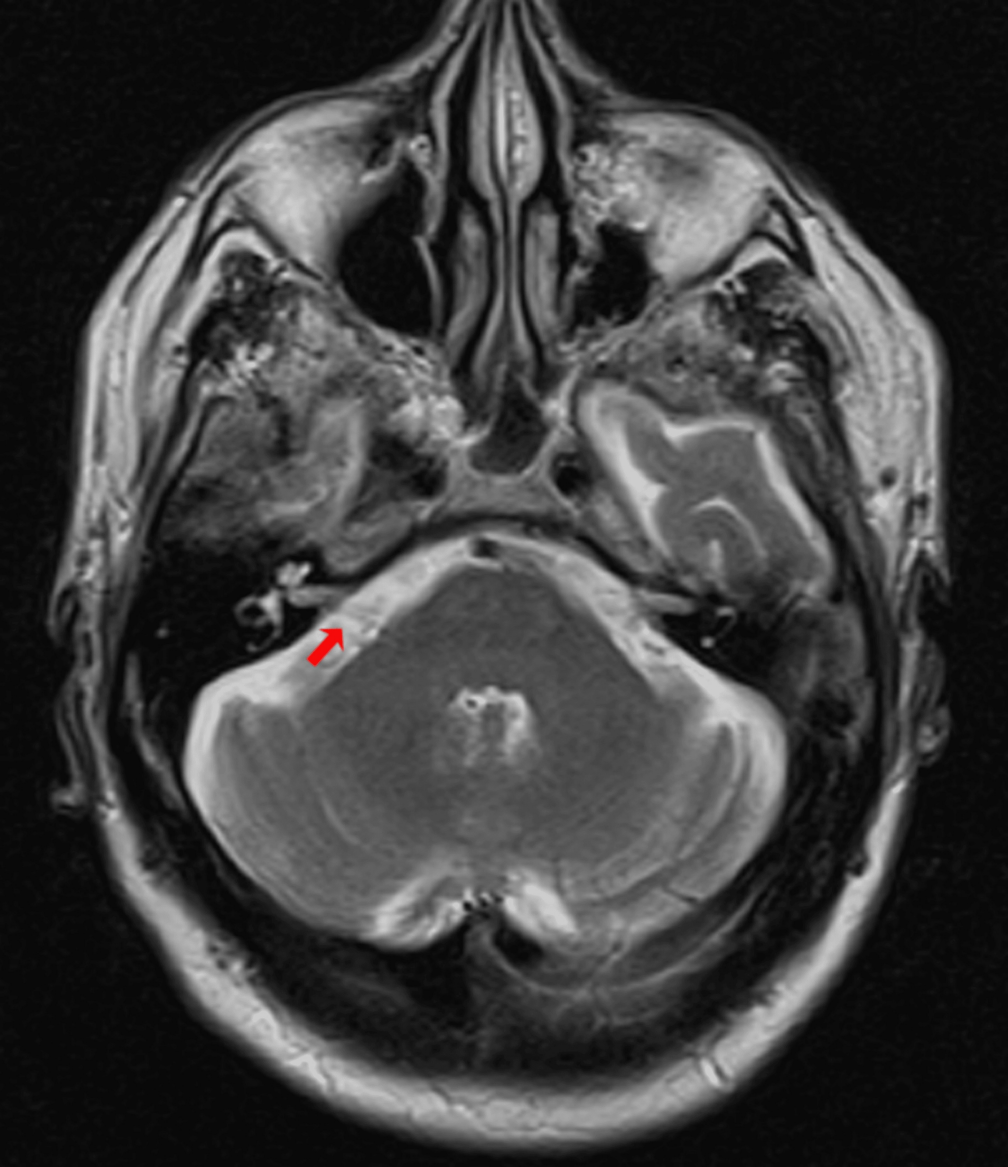
Axial T2-weighted brain MRI of patient no.3 Red arrow: Right-sided neurovascular compression (Chavda classification grade I)

A 73-year-old man attended the audiology and otoneurology department with complaints of two years of low-pitched persistent tinnitus in both ears, bilateral aural fullness that responds to Valsalva maneuvers, and progressive bilateral hearing loss. Non-rotational vertigo attacks lasting seconds related to head movements or exacerbated when walking, and nausea or vomiting were denied. Gastroesophageal reflux, benign prostatic hyperplasia, and long-standing consumption of oral diphenidol history were reported. Tests showed audiometry with bilateral moderate sensorineural hearing loss (Figure 5). Vestibular examination revealed bilateral positional nystagmus mimicking posterior and anterior canal BPPV with no improvement following the Epley and Yacovino maneuvers. The brain MRI reported right neurovascular crossing at the cerebellopontine angle without entering the IAC (Chavda classification grade I) (Figure 6). Daily 300 mg oxcarbazepine was prescribed with partial response, so the dosage was increased to 600 mg, and habituation exercises (Brandt and Daroff) were added. After a month of treatment, the patient reported decreased frequency and intensity of vertigo attacks that matched with the vestibular examination that only evoked a mild positional nystagmus during the left Dix Hallpike maneuver. The DHI was administered before starting treatment with oxcarbazepine and again one month later. The patient reported symptom improvement, transitioning from 'severe disability' to 'moderate disability.' The results are displayed in Table 3.

**Table 3:** The DHI scores of patient no.3 DHI: Dizziness handicap inventory

Subscale	Pre-treatment score	Post-treatment score
Functional	32	14
Emotional	26	6
Physical	22	16
Total	80	36
Category	Severe	Moderate

Case four

**Figure 7:**
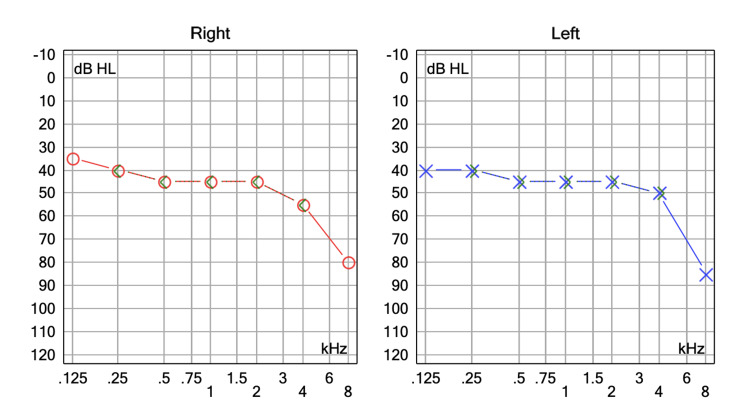
Pure tone audiometry patient no.4

**Figure 8:**
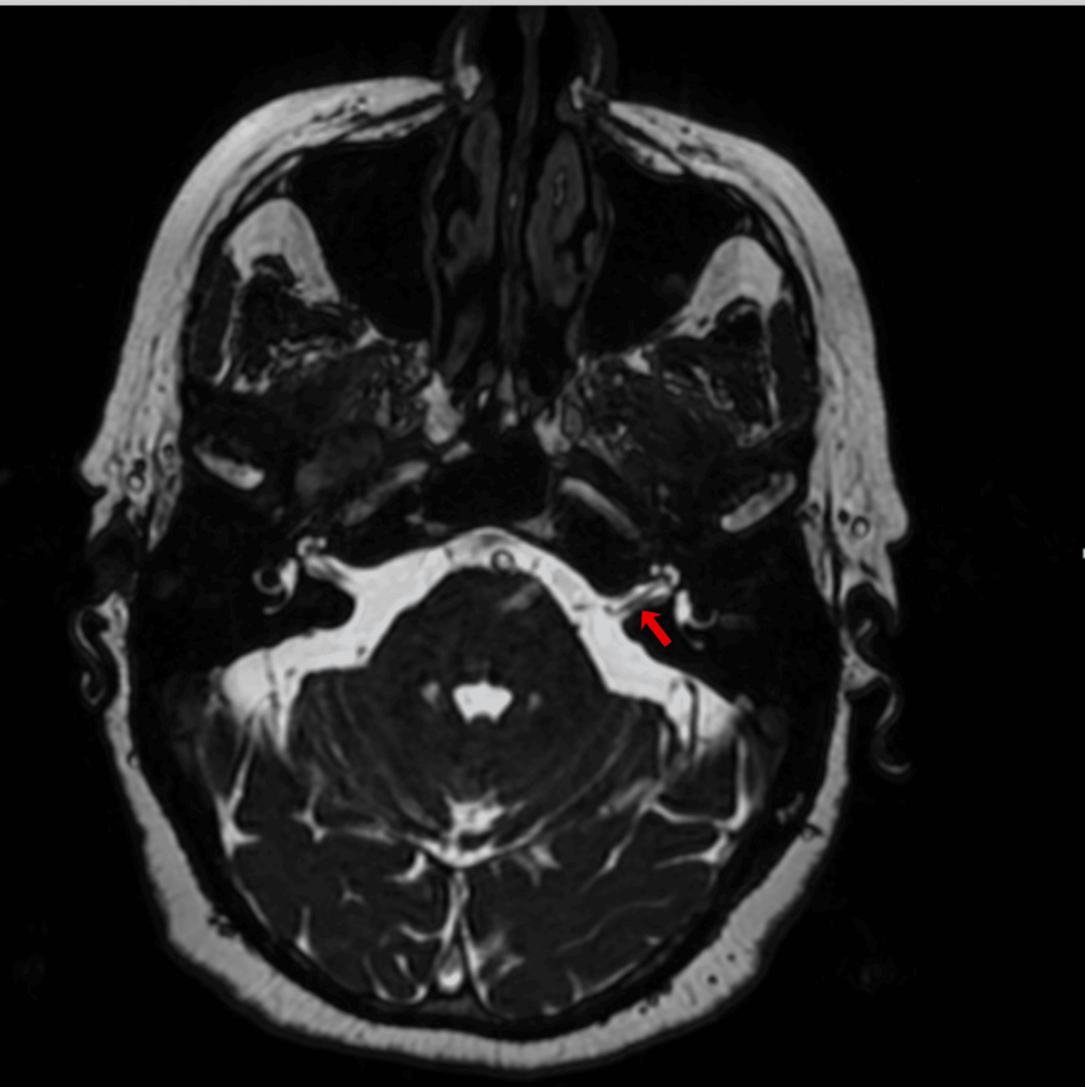
Axial FIESTA brain MRI of patient no.4 Red arrow: Left-sided neurovascular compression (Chavda classification grade III) FIESTA: Fast imaging employing steady-state acquisition

An 85-year-old woman attended the audiology and otoneurology department due to seven-month vertigo attacks lasting one minute, triggered by head movements. She was previously treated with oral cinnarizine and diphenidol, which were discontinued before examination. Under control, arterial hypertension history was reported. Hearing tests revealed bilateral moderate sensorineural hearing loss (Figure 7). The first vestibular examination showed upbeat anticlockwise nystagmus during the right Dix Hallpike maneuver that stopped only after returning the patient to the upright position; an Epley maneuver was done without response. During the follow-up examination, a pure downbeat nystagmus evoked by all positional maneuvers was found. The brain MRI revealed a neurovascular crossing in the left IAC that was classified as Chavda grade III as the vascular loop extends to more than 50% of the IAC (Figure 8). Daily 300 mg oxcarbazepine was prescribed; it reduced the frequency of vertigo episodes as well as their intensity and duration. The DHI was administered before oxcarbazepine treatment and again one month later. The patient’s condition improved from 'severe disability' to 'moderate disability.' The results are presented in Table 4.

**Table 4:** The DHI scores of patient no.4 DHI: Dizziness handicap inventory

Subscale	Pre-treatment score	Post-treatment score
Functional	30	12
Emotional	32	24
Physical	22	10
Total	84	46
Category	Severe	Moderate

## Discussion

Vestibular paroxysmia is classified among neurovascular compression syndromes and is characterized by symptoms resulting from the compression of cranial nerves by vascular loops surrounding them. Within this group, VP is considered a rare syndrome, with limited extensive data available. It originates from the compression of the vestibulocochlear nerve by a vascular loop near the internal auditory canal [7].

The current estimated prevalence of VP among patients with vertigo is approximately 4%. It typically presents in two age groups: one in the early stages of life, often associated with vertebrobasilar abnormalities, and another between ages 40 and 70, which is associated with increased atherosclerosis and arterial hypertension [7]. All the patients described in this report fall into the latter group, being over 40 years old.

The etiology of this syndrome is a chronic compression of the vestibulocochlear nerve. The underlying mechanism appears to involve chronic external pressure on the cranial nerve caused by an adjacent blood vessel loop. This pressure leads to demyelination of the affected area, which lowers the nerve activation threshold and increases its susceptibility to unwanted stimulation through a process known as 'ephaptic transmission' [8].

Neurovascular compression is more commonly observed along the intracisternal segment of the nerve, particularly at the root entry and transition zones, but it can also occur in the meatal zone. The vessels involved include the anterior inferior cerebellar artery (66.7%), the posterior inferior cerebellar artery (13.3%), the vertebral artery (6.7%), the superior cerebellar artery (6.7%), and the vein (6.7%). These percentages are consistent across different studies. Heavily T2-weighted MRI sequences help assess VP [9].

According to the Classification Committee of the Bárány Society, the diagnosis of VP primarily depends on the patient's medical history and the fulfillment of diagnostic criteria. The distinction between definite and PVP is determined by the patient's response to treatment with carbamazepine or oxcarbazepine [5]. We gave a VP diagnosis for each of the patients featured in this case report due to the chronic vestibular syndrome with high frequency and vertigo attacks lasting seconds reported by them, in addition to the vascular loop contacting the VIII cranial nerve revealed by MRI. Contrary to what the consensus document mentions [5], three of the four patients reported vertigo attacks that were triggered by head movements but did not present spontaneously. However, all four patients showed a positive response to oxcarbazepine medication.

A 2019 case-control study found a 100% prevalence of crossed neurovascular compression in patients with VP, compared to 44% in controls (p = 0.0049), which is consistent with previous studies [10-12]. This indicates that VP is likely a true neurovascular compression syndrome, with neurovascular compression on MRI being a sensitive but non-specific finding. Additionally, nerve angulation may be a distinguishing feature [12]. In the current study, all patients with a neurovascular crossing underwent MRI with the FIESTA sequence; however, due to image quality limitations, the angulation observed in some studies could not be analyzed.

Differential diagnoses include various episodic vestibular syndromes such as Meniere’s disease, Tumarkin otolithic crisis (vestibular drop attacks), paroxysmal brainstem attacks with vertigo, dysarthria, or ataxia (following a stroke or in multiple sclerosis), vestibular migraine, vertebrobasilar transient ischemic attacks, panic attacks, perilymphatic fistula, episodic ataxia, epilepsy with vestibular aura, benign paroxysmal positional vertigo, central positional vertigo, rotational vertebral artery occlusion syndrome (RVAOS), orthostatic hypotension, and rarely, cerebellopontine angle cysts or tumors [5]. None of these conditions better accounted for the clinical features presented by our patients.

For all patients with PVP, a therapeutic trial with low-dose carbamazepine (200 to 800 mg/day) or oxcarbazepine (300 to 900 mg/day) is recommended. A positive response to these medications supports a definitive diagnosis. If patients are intolerant, alternative treatments with phenytoin or valproic acid may be considered; however, further research is needed [5]. In the four patients presented in this report, treatment with oxcarbazepine was effective. Two of them experienced drowsiness, leading to changes in their sleep patterns, such as falling asleep earlier or waking up later. However, these adverse effects were manageable and tolerable.

For follow-up, we should consider that approximately 20% of patients may experience complete remission without medication, so a gradual reduction in it could be evaluated in selected cases. Since most patients report satisfactory long-term responses with medication, surgical microvascular decompression should be reserved for cases that are truly refractory [4].

A new treatment option under investigation is lacosamide, initially administered at 50 mg twice a day with dose adjustments based on efficacy. Lacosamide is associated with fewer side effects compared to carbamazepine and oxcarbazepine, which may benefit patients who cannot tolerate these medications, with discontinuation rates around 60% [13]. The adverse effects of oxcarbazepine experienced by our patients were well tolerated. If adverse effects increase or if there is a decrease in response to oxcarbazepine, transitioning to lacosamide should be considered.

## Conclusions

According to the reports presented, VP presents significant diagnostic challenges due to its symptoms resembling other vestibular conditions, particularly BPPV, as both can involve attacks triggered by head movements. While a detailed medical history is crucial, MRI plays an indispensable role in confirming neurovascular compression. All patients reported here had previously undergone unsuccessful treatments before receiving the correct diagnosis, which was ultimately confirmed by their positive response to oxcarbazepine. This aligns with the diagnostic criteria established by the Bárány Society. Although oxcarbazepine proved highly effective in reducing the intensity and frequency of vertigo attacks, some patients experienced side effects, such as drowsiness. Alternatives, such as lacosamide with fewer side effects, are available but require further study. Surgical interventions, like microvascular decompression, should be considered for patients who do not respond to medication. In some cases, patients may experience spontaneous remission, suggesting that long-term treatment plans should consider gradual medication reduction when appropriate. Raising awareness of VP is critical to ensuring timely diagnosis and intervention, as many patients face a prolonged disease course before receiving appropriate treatment. Early and accurate diagnosis, paired with effective management, can significantly improve the patient’s quality of life.
